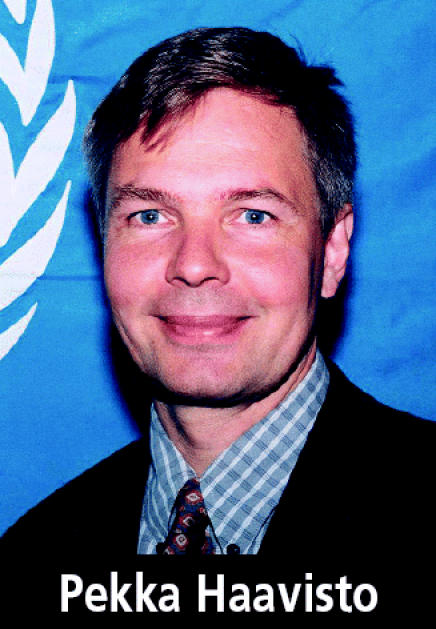# Guest Editorial: The Legacy of Conflicts

**DOI:** 10.1289/ehp.112-a976

**Published:** 2004-12

**Authors:** Pekka Haavisto

**Affiliations:** United Nations Environment Programme, Geneva, Switzerland, E-mail: pekka.haavisto@unep.ch

The pictures during wars and conflicts usually bring to our living rooms the misery of human suffering. When conflicts are over, the news usually concentrates on reconstruction issues. However, the legacy of conflicts might also include longer-term security threats, such as environmental degradation, damaged infrastructure, and conflict-related risks to human health. Because of poor environmental administration in postconflict countries, environmental and health risks may not be addressed properly.

For the past 5 years the United Nations Environment Programme (UNEP) has been working in countries where the natural and human environments have been damaged as a direct or indirect consequence of conflict. In 1999, as the ruins of targeted industrial facilities in Kosovo, Serbia, and Montenegro were still smoldering, UNEP teams conducted the first postconflict environmental assessment there.

UNEP postconflict environmental assessments seek to identify immediate risks to human health and livelihoods and provide recommendations on priorities for clean-up, sustainable use of natural resources, and for strengthening environmental governance.

In the Balkans UNEP concluded that there were several environmental hot spots—such as targeted industrial facilities and oil refineries in Pancevo, Novi Sad, Kragujevac, and Bor—where immediate cleanup was needed to avoid further threats to human health. The Danube was at risk because of leakage of more than 60 different chemicals, including mercury, from Pancevo. These findings led the international community for the first time to include environmental clean-up in their postconflict humanitarian aid.

Since then, UNEP has conducted postconflict activities in Afghanistan, Bosnia and Herzegovina, Iraq, Liberia, the Occupied Palestinian Territories, and Serbia and Montenegro.

UNEP’s report on Afghanistan’s postconflict environmental assessment identified the pressures on the natural resources, including waters, soils, forests, and wildlife, and linked poor environmental management in the waste and water sectors directly to human health risks (UNEP 2003a). UNEP found that most of the country is subject to an alarming degree of environmental degradation exacerbated by poverty and population growth. Moreover, many of Afghanistan’s environmental problems can be traced back to the collapse of local and national forms of governance and resource management, highlighting the urgent need to rebuild the Afghan environmental administration.

In early 2003 UNEP published a *Desk Study on the Environment in Iraq* (UNEP 2003b). The report provided a timely overview of key environmental issues in the context of the recent conflict in Iraq. It also took into consideration the chronic environmental stress already in place from the Iran–Iraq war of the 1980s, the 1991 Gulf War, the unintended effects of the UN sanctions and environmental mismanagement by the former Iraqi regime. For example, draining the Mesopotamian Marshes and building artificial waterways has ruined some of the most valuable areas of biodiversity in Iraq. The water pollution is affecting not only the Euphrates and Tigris Rivers but also the wider Persian Gulf region.

The *Desk Study on the Environment in the Occupied Palestinian Territories* (UNEP 2003c) identified acute environmental problems that have arisen as a result of the ongoing conflict, as well as problems stemming from long-term inadequate resource allocation and environmental management. The report concluded that, despite the current political difficulties, environmental problems should be addressed immediately in order to preserve natural resources and establish a safe environment for future generations.

Wherever there has been a conflict, there are also environmental consequences. On the African continent UNEP has been working in Liberia, where the misuse of natural resources has not only been a source of conflict but has also sustained it. Furthermore, one of the most severe consequences of the conflict has been the massive movement of refugees and internally displaced people. A key contribution toward increasing regional stability will be to provide the Liberian government and people with the capacity and proficiency to manage their natural resources in a just and sustainable manner. Now the international community has to ensure that environmental issues are fully integrated into the overall reconstruction efforts.

Based on UNEP experience, there are certain general recommendations that can be made, despite the uniqueness of every postconflict situation. First, the environment cannot wait. Environmental experts should enter the country as soon as possible after the conflict to facilitate a proper assessment and integration of environmental issues into humanitarian aid and reconstruction efforts.

Second, support and capacity building of the existing or newly established environmental administration is crucial for long-term sustainability. Third, many postconflict countries have been suffering from political isolation, and there is an urgent need to reintegrate them into regional and international environmental cooperation.

The rules of warfare have been widely debated since the global war on terrorism started. What are the humanitarian principles that should be followed? Or are we adopting new rules in a new situation? Also the environmental rules of warfare should be debated. The ENMOD convention (UN 1977) already forbids environmental modification as a part of warfare: man-made floods or earthquakes are not allowed as weapons in wars.

Because targeting industrial facilities or using different type of weapons can pose high risks for populations, we should open a debate about the environmental rules of modern warfare. If there are wars, there must be rules.

## Figures and Tables

**Figure:**